# Retraction notice for: “Kaempferol suppresses human gastric cancer SNU-216 cell proliferation, promotes cell autophagy, but has no influence on cell apoptosis” [Braz J Med Biol Res (2019) 52(2): e7843]

**DOI:** 10.1590/1414-431X20207843retraction

**Published:** 2020-08-03

Fan Zhanghttps://orcid.org/0000-0002-1084-1183^1^ and Cuimei Mahttps://orcid.org/0000-0002-8817-0789^2^

^1^Teaching and Research Department of Diagnostics, Jining Medical University, Jining, China

^2^Department of Gastroenterology, Affiliated Hospital of Jining Medical University, Jining, China

Correspondence: Cuimei Ma: <cuimeima1@sina.com>

**Retraction for:** Braz J Med Biol Res | doi: http://10.1590/1414-431X20187843 | PMID: 30785478 | PMCID: PMC6376319

The Brazilian Journal of Medical and Biological Research received a request from the authors to withdraw this manuscript. Meanwhile, the Editors became aware of a denouncement published by independent journalists from the “For Better Science” website including this paper. This denouncement consisted of potential data falsification and/or inaccuracy of results in western blots and flow cytometry plots.

As per consensus between the Authors and the Editors-in-Chief of the Brazilian Journal of Medical and Biological Research (BJMBR), the article titled “Kaempferol suppresses human gastric cancer SNU-216 cell proliferation, promotes cell autophagy, but has no influence on cell apoptosis” that was published in year 2019, volume 52, issue 2, (Epub Feb 14, 2019) has been retracted.

